# Optimization of diagnosis and treatment of hematological diseases via artificial intelligence

**DOI:** 10.3389/fmed.2024.1487234

**Published:** 2024-11-07

**Authors:** Shi-Xuan Wang, Zou-Fang Huang, Jing Li, Yin Wu, Jun Du, Ting Li

**Affiliations:** ^1^The Endemic Disease (Thalassemia) Clinical Research Center of Jiangxi Province, Department of Hematology, First Affiliated Hospital of Gannan Medical University, Ganzhou, China; ^2^The Third Clinical Medical College of Gannan Medical University, Ganzhou, China; ^3^Department of Hematology, School of Medicine, Renji Hospital, Shanghai Jiao Tong University, Shanghai, China

**Keywords:** artificial intelligence, machine learning, deep learning, precision medicine, diagnosis, treatment, Hematology

## Abstract

**Background:**

Optimizing the diagnosis and treatment of hematological diseases is a challenging yet crucial research area. Effective treatment plans typically require the comprehensive integration of cell morphology, immunology, cytogenetics, and molecular biology. These plans also consider patient-specific factors such as disease stage, age, and genetic mutation status. With the advancement of artificial intelligence (AI), more “AI + medical” application models are emerging. In clinical practice, many AI-assisted systems have been successfully applied to the diagnosis and treatment of hematological diseases, enhancing precision and efficiency and offering valuable solutions for clinical practice.

**Objective:**

This study summarizes the research progress of various AI-assisted systems applied in the clinical diagnosis and treatment of hematological diseases, with a focus on their application in morphology, immunology, cytogenetics, and molecular biology diagnosis, as well as prognosis prediction and treatment.

**Methods:**

Using PubMed, Web of Science, and other network search engines, we conducted a literature search on studies from the past 5 years using the main keywords “artificial intelligence” and “hematological diseases.” We classified the clinical applications of AI systems according to the diagnosis and treatment. We outline and summarize the current advancements in AI for optimizing the diagnosis and treatment of hematological diseases, as well as the difficulties and challenges in promoting the standardization of clinical diagnosis and treatment in this field.

**Results:**

AI can significantly shorten turnaround times, reduce diagnostic costs, and accurately predict disease outcomes through applications in image-recognition technology, genomic data analysis, data mining, pattern recognition, and personalized medicine. However, several challenges remain, including the lack of AI product standards, standardized data, medical–industrial collaboration, and the complexity and non-interpretability of AI systems. In addition, regulatory gaps can lead to data privacy issues. Therefore, more research and improvements are needed to fully leverage the potential of AI to promote standardization of the clinical diagnosis and treatment of hematological diseases.

**Conclusion:**

Our results serve as a reference point for the clinical diagnosis and treatment of hematological diseases and the development of AI-assisted clinical diagnosis and treatment systems. We offer suggestions for further development of AI in hematology and standardization of clinical diagnosis and treatment.

## 1 Introduction

During the past decade, comprehensive clinical diagnosis and treatment of hematological diseases have become increasingly challenging. Hematological diseases are numerous and complex, and include leukemia, lymphoma, myelodysplastic syndrome (MDS), multiple myeloma (MM), and myeloproliferative neoplasms. In the realm of hematological disorders, a thorough diagnostic approach frequently integrates morphological assessment, immunological evaluation, cytogenetic analysis, and molecular biological techniques. While these diagnostic modalities are indeed effective, they impose significant demands on the clinicians’ expertise, detection methodologies, and experiential knowledge. Furthermore, the accuracy of test results improves with the number of samples, requiring substantial infrastructure investment. This makes it difficult to achieve full coverage in rural or underdeveloped areas, creating an uneven healthcare system. Most hematological diseases are refractory, and traditional clinical treatments such as general supportive therapy, immunotherapy, and radiotherapy are tailored to different diseases and systemic symptoms. In addition, it is often challenging to achieve high prognostic accuracy. Although new treatment options have been introduced in recent years, including targeted therapies, immunotherapy, and hematopoietic stem cell transplantation, some patients still experience poor outcomes and face potential drug resistance, post-transplantation relapse, and related complications.

Artificial intelligence (AI) represents a sophisticated technical solution that emulates human cognitive functions, whereas machine learning (ML) and deep learning (DL) are specialized subsets of AI dedicated to developing software systems capable of learning from data and enhancing their performance accordingly. Machine learning, which enables computers to derive insights from data without the need for explicit programming, serves as the foundational methodology for endowing machines with human-like intelligence. With the accumulation of massive datasets and the rapid development of AI, the medical research field is increasingly embracing the “AI + medical” model. AI algorithms, including DL, support vector machine (SVM), random forest (RF), genetic algorithms, and natural language processing (NLP), enable computers to ‘learn” (1). This allows them to quickly and accurately process vast amounts of medical data from different diagnostic modalities. AI has been widely applied to hematology-related morphological testing, immunological testing, chromosome karyotype analysis, gene sequencing, biomarker identification, drug development, risk stratification, and prognosis monitoring.

In recent years, advances AI technology have led to AI models that can achieve high efficiency and accuracy, comparable to or even surpassing human experts. In addition, the lower marginal cost of AI (the cost per additional sample after system training) and its ability to process massive amounts of data indicate its potential to build data analytics models that can be remotely accessed and dynamically tracked (2). This capability can optimize medical treatment protocols, personalize clinical treatment guidance, and help medical facilities to accurately allocate healthcare resources, thereby promoting the precision treatment of hematological disorders.

Several key scientific advances in AI have been made in regard to the diagnosis and treatment of hematological diseases. For example, in the comprehensive typing diagnosis, AI-assisted detection systems such as ML-based CellaVision (FDA approved) (3), Scopio Labs X100 (FDA approved) (4), Techcyte (5), and Morphogo (6) have been developed to automate morphological identification, labeling, counting, and analysis of bone marrow smears and peripheral blood smears. The integration of multi-algorithmic DeepFlow (7–9) has streamlined multiparameter flow cytometry (FC), optimizing the process of immunological diagnosis. AI systems for automatic karyotype analysis in cytogenetics based on convolutional neural networks (CNNs), such as Varifocal-Net (10) and KaryoNet (11), have improved on issues such as the low resolution of karyotypic maps and the difficulty detecting cryptic chromosomal abnormalities (12–14).

Using DL algorithms and high-throughput sequencing technology, the potential heterogeneity of blood diseases can be mined in genomics, transcriptomics, and proteomics, particularly in the diagnosis of leukemia. In addition, the newly developed chromatin interaction neural network (ChINN) overcomes limitations in regional genomic testing (15). Numerous clinical applications have shown that AI systems are very effective for assisting medical diagnosis, with high productivity and accuracy comparable to experienced physicians. However, manual assessment by an expert is still essential for disease reclassification, validation, and interpretation of results.

ML has demonstrated promising results in the personalized and precise treatment of hematological diseases. It can use clinical data to build data analytics models for disease prediction, risk stratification, and the tailoring of optimal treatment plans based on each patient’s individual characteristics and condition (16–19). Furthermore, it can assist in mining new drug therapy targets or pathways (20), promoting the innovative development of the clinical treatment of hematological diseases.

However, there are challenges in applying AI technology, such as the lack of access standards for AI products, standardized data, medical–industrial collaboration, privacy and ethical issues, and the “black-box” nature of algorithms. Therefore, the regulation of AI products should be prioritized, along with addressing technical shortcomings and continuously strengthening education and training in the interdisciplinary field of AI and medicine. This is particularly important for clinical hematologists and undergraduate medical students.

During the past 5 years, the outbreak of COVID-19, the surge in the prevalence of hematological malignancies, and the globalization of the epidemic have caused significant harm to humanity. These challenges have also prompted consideration of AI-assisted diagnosis and treatment. There is a pressing need for reliable methods with high efficiency, high precision, and low cost to enhance the diagnostic and therapeutic environment for hematological diseases.

**Figure 1:**
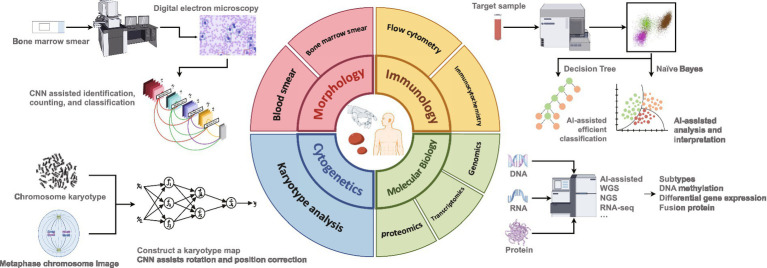
Assistance of artificial intelligence in comprehensive diagnosis of hematological diseases.

Here, we review the application of AI in hematology diagnosis and treatment over the past 5 years, with a particular focus on comprehensive typing diagnosis (including morphology, immunology, cytogenetics, and molecular biology) (Figure 1) to clarify the role and limitations of AI in areas such as disease prediction, drug development, risk stratification, and prognosis tracking. We speculate on how the integration of AI algorithms in the future will optimize the intelligence, standardization, and consistency of routine hematology diagnosis and treatment. The aim is to provide uniform and standardized treatments for patients with specific hematological disorders, reduce variation in patient outcomes due to differences in medical practice, and significantly improve the efficiency of diagnosis and treatment and the integration of medical resources.

## 2 Application of AI in the diagnosis of hematological disease

### 2.1 AI-assisted morphological diagnosis of hematological disease

Hematological disorders are a complex group of diseases for which early diagnosis is crucial for effective clinical management. Cytomorphometric testing of bone marrow smears is one of the most common and effective diagnostic methods for blood diseases in clinic. Traditional manual analysis of smears begins with a hematologist selecting an area of interest with an appropriate distribution of cell trajectories and then performing a differential cell count on hundreds of cells to identify various cellular subsets and subtypes. This process is labor-intensive, cumbersome, and time-consuming (21). Because of the inherent complexity of bone marrow aspirate (BMA) samples, diagnosis largely depends on the hematopathologist’s experience, which makes the results highly subjective (22). In recent years, a promising technology has emerged to improve the accuracy and efficiency of morphological diagnosis: the combination of AI ML and digital morphological pathology analysis (Table 1). This approach aids diagnosis, with CNNs being particularly effective for the automated morphological detection and typing of BMA samples. CNNs are widely used for this purpose (23), and those enhanced with the CatBoost and XGBoost algorithms are particularly optimal (24).

**Table 1:** Application of artificial intelligence in morphological aspects of hematology diagnosis.

Diseases	System	Purpose and effect	AI	References
AA, MDS, AML	Recognition model constructed by image-net pre-trained model	Automatic differentiation of AA, MDS and AML based on bone marrow smears	CNN	(28)
APL	Mask R-CNN	Detection and classification of nucleated cells using example segmentation methods	CNN	(26)
MDS	DenseNet, YOLO	Detection and classification of cellular and non-cellular objects in samples	CNN	(140)
MDS	BMSNet	Evaluation of single-nucleated sphere morphology in bone marrow smears	CNN	(29)
Leukemia	YOLOX-s, MLFL-Net	Cellular detection, classification and prediction of leukemia types	CNN	(30)
Leukemia	CNN Model	Recognize all subtypes of leukemia	CNN	(32)
Leukemia	ANN + FFNN+SVM^1^	Early detection of leukemia	CNN	(33)
	AlexNet, GoogleNet, ResNet-18^2^			
	CNN-SVM^3^			
AML	FRCNN, VGG Image Annotator, ENN, Xception CNN, ResNet50	Distinguish AML and predict the mutational status of NPM1	CNN	(34)
Leukemia	Faster R-CNN	Automatically detect bone marrow cells and determine their type	R-CNN	(25)
AML, MM	BMAsDCC	Detect and classify all non-neoplastic bone marrow cell components of DCC and tumor cells	VGG16 CNN	(22)
APL	The multi-stage DL platform	Automatically reads bone marrow smear images, accurately segments cells, predicts APL	Xception CNN	(27)
AML, ALL, CML, CLL	ResNet50	Automated analysis of bone marrow smears using only slide-level labels	DL	(141)
HD	AI-assisted Digital DCC System	BMA analysis for cell type counting and differentiation in an efficient and objective manner	DL	(31)
Leukemia	Techcyte	WBC identification and vesicle recognition	DL	(37)
HM	Techcyte	Assessing the accuracy of WBC classification and primitive cell identification	DL	(5)
PBS	Scopio Labs X100	Scanning of peripheral blood smears and BMA samples	DL	(4)
MPN	Single Shot Multibox Detector	Determine megakaryocyte cytomorphologic subtypes and correlate extracted features with potential diagnosis of MPN or reactive/non-tumor mimics	DL	(38)
ALL	ALL Detector (ALLD)	Distinguishing ALL patients based on primary cellular micrographs	DL	(142)
BMA	Morphogo	Automated cell sorting of bone marrow cells	ML	(6)
MCBM	Morphogo	Identifying metastatic atypical cancer clusters and facilitating rapid diagnosis	ML	(143)

AA, aplastic anemia; AI, artificial intelligence; ALL, acute lymphoblastic leukemia; AML, acute myeloid leukemia; APL, acute promyelocytic leukemia; ANN, artificial neural network; BMA, bone marrow aspiration; CLL, chronic lympholeukemia; CNN, convolution neural network; DCC, differential cell count; DL, deep learning; ENN, ensemble neural network; FFNN, feedforward neural network; FRCNN, faster region convolutional neural network; HD, hemolymph diseases; HM, hematological malignancies; MCBM, metastatic cancer of bone marrow; MDS, myelodysplastic syndrome; ML, machine learning; MM, multiple myeloma; MPN, myeloproliferative neoplasms; NPM1, nuclear phosphoprotein 1; SSD, single-session detection; PBS, peripheral blood smear; SVM, support vector machine; WBC, white blood cells.

**Figure 2:**
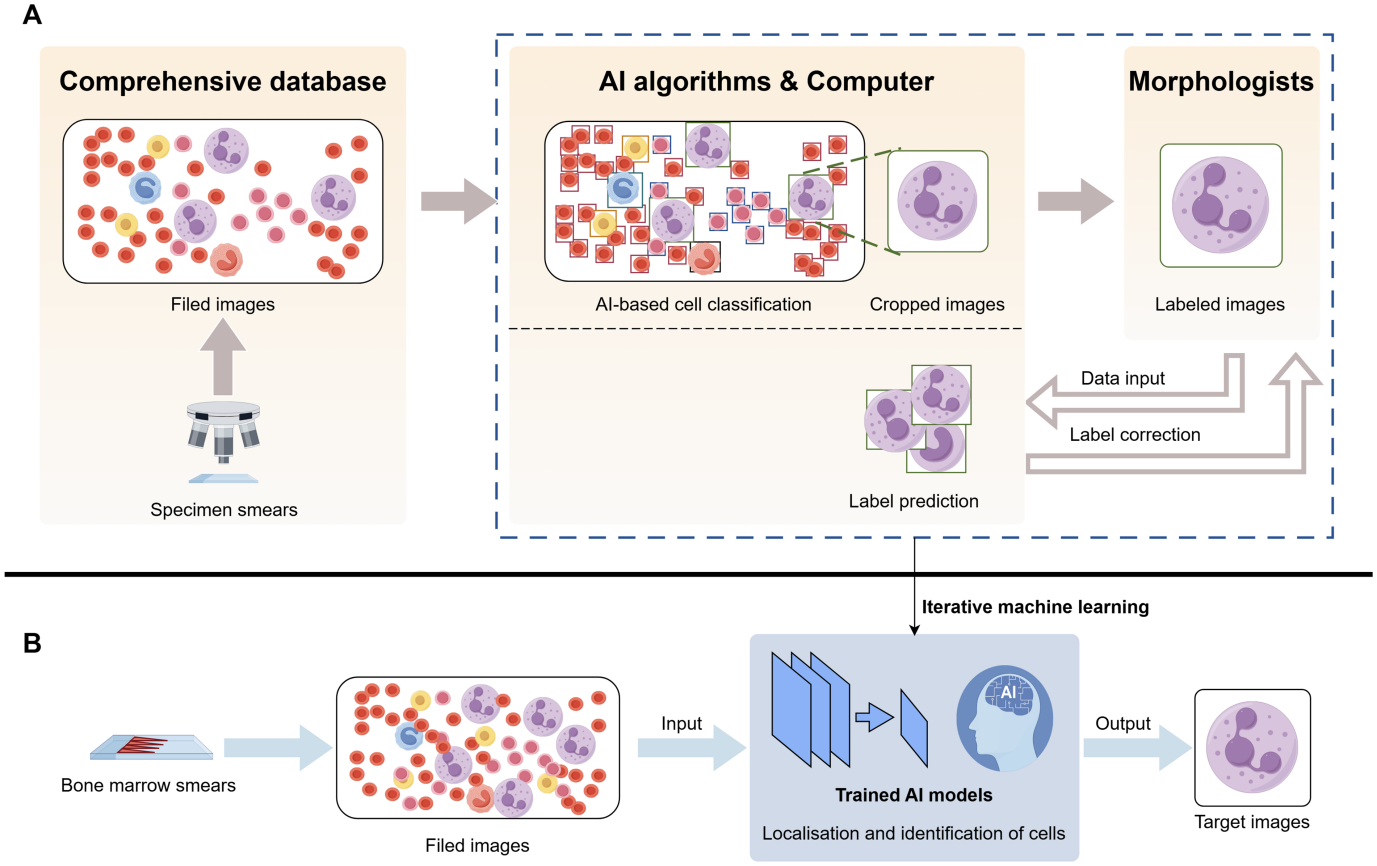
Artificial intelligence identifies neutrophils in bone marrow smears. (A) Bone marrow smears were obtained from a database and raw images were acquired, the raw images were located and identified by a computer AI algorithm to crop out the neutrophils and reviewed by a morphologist and corrected in several iterations. (B) After obtaining raw bone marrow smears, selecting images, and feeding them into an artificial intelligence model trained by iterative machine learning, the target cell neutrophils are output.

The powerful role of CNN in automatic analysis of bone marrow smear data has been applied to a variety of blood diseases, such as aplastic anemia (AA), MDS, AML, etc. (25–28). The CNN-based you only look once (YOLO) model is a target-detection algorithm developed to rapidly and accurately detect and classify individual target cells in bone marrow smears (Figure 2). As early as 2020, this algorithm was used to evaluate individual cells in bone marrow smears of patients with MDS morphology (29). In order to improve accuracy, Wang et al. (30) developed a new YOLOX-s model. By combining MLFL-Net, a new architecture with multi-level features, the total accuracy was as high as 89.53%, and the diagnosis and prediction of acute leukemia also reached 92.5% of the cohort (compared with manual experts), which was better than all other relevant models. In 2024, a significant development in microscopy digital systems emerged with a single-trial detection architecture and the MobileNet V2 backbone. The most important innovation of this system is its ability to overcome the limitations of traditional AI-based BMA analysis systems, such as the inability to analyze and share data remotely and issues with low reproducibility. This new system facilitates human–computer interaction in any hospital through an intelligent BMA digital platform, eliminating the need for specific and complex medical electronic devices (31). This advancement strongly supports the feasibility of AI in enhancing the diagnosis of clinical hematological disease.

In the context of identifying leukemia subtypes, CNNs outperform other ML algorithms, including plain Bayes, SVMs, k-nearest neighbor, and decision tree algorithms (32). Newer algorithms, such as Cat-Swarm Optimization, further optimize CNN performance by combining them with CNN architectures. Current research favors either highly accurate single-algorithmic systems or systems that combine multiple algorithms for early detection of leukemia. For example, artificial neural networks, feed-forward neural networks, AlexNet + SVM, and ResNet-18 + SVM can all achieve 100% accuracy in leukemia diagnosis (33). These systems not only reveal morphological features but also predict the mutation status of genes in leukemia, such as the *NPM1* mutation, common in AML (34). The high numerical accuracy of CNN model applications highlight the promise of more efficient and accurate hematological diagnoses in the clinical setting. This indicates a new direction for subsequent research to continue refining and optimizing CNN models for optimal clinical integration.

Morphological diagnosis in hematopathology also relies heavily on the analysis of peripheral blood smears. However, current digital cell imaging systems can only analyze limited areas of these smears and require manual intervention. With the continuous advancement of ML, more applications in hematological digital pathology can now extract and aggregate information from multiple data sources, including peripheral blood smears. These applications support diagnosis by simulating manual recognition workflows and thought modeling, thus enhancing the diagnostic process through improved accuracy and efficiency (35). Previous studies have developed advanced algorithms such as CellaVision and computer-assisted peripheral blood smear analysis for diagnosis. These technologies expand the hematopathologist’s capabilities, dramatically streamline workflow, and reduce turnaround time. By automating the analysis process and providing rapid, accurate results, these systems help enhance the efficiency and accuracy of hematological diagnoses (3). For example, the Scopio Labs X100 (Scopio Labs; Tel Aviv-Yafo, Israel) is an AI-based digital microscope imaging system that uses a full-field view approach to localize and classify blood cells in peripheral blood smears, marking a significant achievement by integrating a digital system with a microscope (4). Its digital nature allows for remote viewing by clinicians (36). Techcyte (Techcyte Inc., Orem, UT, United States) also supports remote viewing (5). These systems drastically reduce turnaround time for clinical work and significantly improve the efficacy of identifying malignant cells in leukemia over time (37). Compared to traditional morphological analysis, a fully automated ML pipeline can more rapidly provide an objective and accurate initial differential assessment of peripheral blood smears. This capability is particularly valuable for regions or institutions with limited healthcare resources and specialized hematopathology talent (38).

### 2.2 AI-assisted immunophenotypic diagnosis of hematological disease

In the clinical setting, FC and multiparameter FC (MFC) are essential auxiliary methods for examining the bone marrow of patients with hematological disease (39). These technologies are a class of high-throughput and high-sensitivity detection technologies that use a combination of different fluorescently labeled antibodies to detect the expression of antigens on the surface or intracellular of hematopoietic cells, and then analyze and determine the serial origin, differentiation degree, and abnormal phenotypic carnivore of cells. They have become an essential tool in the diagnosis and typing of blood diseases. However, traditional FC analysis relies on manual interpretation by the analyzer, which is time-consuming and prone to errors. The sensitivity and accuracy of these tests are subjective and depend significantly on the analyzer’s experience. Continuous exploration and development in recent years have led to computational analysis methods combined with AI, which has achieved promising results. These AI-enhanced methods have improved the accuracy and efficiency of the diagnostic process. In addition to identifying and classifying cells, DL-based AI models can reveal new biological associations between FC marker expression and cytogenetic and molecular variants in hematological disorders (40), providing clinicians with various differential diagnoses (39). Furthermore, they enhance the accuracy and standardization of clinical diagnoses through advanced visualization and automated gated analysis techniques (41).

However, the MFC data for most AI models are limited by specific panels and are often idiosyncratic. Therefore, DeepFlow, a clinically generalized FC panel based on the multidimensional density-phenotype coupling algorithm, has been developed in recent years (7–9). It dynamically adapts to changes in reagents and instrument settings, reducing the time required to accurately cluster and analyze cell lineages to less than 5 min (42). In addition, the accuracy of the results of leukemia classification and diagnosis using DeepFlow in combination with unsupervised learning algorithms, multidimensional clustering algorithms, and RF is almost identical to that of manual analysis (9). This leads to faster determinations and more accurate classifications of lymphocyte subpopulations, compensating for the deficiencies of traditional techniques and improving quality and standardization. The integration of AI and ML is expected to address healthcare disparities by enhancing the precision and consistency of immunological diagnostic processes, thus providing more equitable healthcare solutions (42). However, this does not mean that the role of humans will be completely replaced by AI. Instead, AI plays a crucial role in providing objective and standardized clinical advice to assist healthcare professionals.

Immunohistochemistry (IHC) represents a sophisticated immunological detection technique that aids in the diagnosis of hematological disorders. However, the rapid growth of IHC data makes manual interpretation of IHC data inefficient. In order to solve this problem, an artificial intelligence system based on Bayesian theorem probabilistic decision tree algorithm, ImmunoGenius, has been developed. It can be applied to mobile platforms such as iOS and Android platforms to diagnose and predict B-cell lymphoma and T-cell lymphoma, and provide clinical hematopathologists and hematologists with more convenient interpretation of IHC. However, due to the lack of specific IHC markers and overlapping IHC profiles in the clinical detection of AI systems, it is still necessary to combine clinical artificial and other diagnostic information such as histology (43–45) (Table 2).

**Table 2:** Application of artificial intelligence in immunology in the diagnosis of hematological diseases.

Disease	System	Immunization information	Function	AI algorithm	References
Blood immunodeficiency diseases	DeepFlow	T, B, and NK cells and important subsets of immune cells	Improve the accuracy and efficiency of flow cytometry in efficiently diagnosing immune diseases	MDPC	(42)
Hemolymph neoplasms	ImmunoGenius	IHC results for lymphoid tumors	Auxiliary diagnose lymphoma	Probabilistic decision tree algorithm based on Bayes theorem	(44)
Hemolymph neoplasms	ImmunoGenius	IHC profiles of 2009 antibodies	Provide a second opinion of the IHC interpretation to support the pathological diagnosis	Probabilistic decision tree algorithm based on Bayes theorem	(45)
Hemolymph neoplasms	ImmunoGenius	IHC data for 150 lymphoid tumors and 584 antibodies	Diagnosis of lymphoid tumor	Probabilistic decision tree algorithm based on Bayes theorem	(43)
MDS	Flow cytometry diagnostic system for MDS	Six tubes based on FC panel	Distinguish between MDS and non-neoplastic cytopenia	FlowAI, FlowSOM, Random Forest	(144)
AML	ABMILM	1,820 flow cytometry samples	Automatic diagnosis of AML and molecular characterization	DL	(40)
AML	Cross-panel sample-level classification model	FC data from bone marrow aspirate samples	Diagnosis and classification of AML	GMM-SVM	(41)

IHC, immunohistochemistry; AML, acute myeloid leukemia; DL, deep learning; MDS, myelodysplastic syndromes; GMM-SVM, Gaussian mixture model support vector machine; MDPC, multidimensional density-phenotype coupling.

### 2.3 AI-assisted cytogenetic karyotyping for diagnosis of blood disorders

Hematological diseases exhibit a wide range of cytogenetic abnormalities, with significant variability even among patients with the same condition. Hematological cancer cells often display multiple aberrations such as rearrangements, deletions, and duplications. Correct interpretation of these abnormalities is crucial for disease classification, prognostic determination, and therapeutic decision-making in patients with hematological malignancies.

Despite improved understanding of hematological malignancies due to increased genetic data availability, applying these large, complex, and time-consuming datasets in clinical practice remains challenging. Chromosomal karyotyping is one of the most important diagnostic tools in hematological cytogenetic laboratory tests. Typically, pathologists manually classify and construct karyotype maps of G-banded metaphase chromosomes based on each chromosome’s specific length and banding pattern. However, this is both costly and inefficient.

Over the years, ML methods have shown great potential for automating the analysis of large amounts of cytogenetic data (Table 3). To increase the rate of chromosome classification and karyotyping, various AI systems for automated mid-cycle capture, semi-automated, or interactive karyotyping have been introduced, leading to progressively wider clinical applications. These AI-driven systems enhance efficiency and accuracy, reducing the time and costs associated with traditional manual chromosomal karyotyping (46, 47). Using the CNN model to correct rotation and position in conventional karyotype analysis, the accuracy of identifying and classifying intermediate chromosomes of fluorescent R-bands could be improved to 98.8% (48). The accuracy of R-band and G-band chromosome classification can be improved by training with the characteristics of intra-karyotype interaction and category distribution through ML, which provides a new idea for accurate karyotype analysis of patients with different types of numerical abnormalities (11).

**Table 3:** Application of artificial intelligence in chromosome karyotype analysis of blood diseases.

Researcher	Year	System	Input	Function	AI	References
Qin	2019	Varifocal-Net	Chromosome karyotype map	Classification of chromosome types and polarity	CNN	(10)
Vajen	2022	CNN Model	Fluorescent R-band metaphase chromosomes	Identify chromosomes in cancer cells	CNN	(48)
Bokhari	2022	ChromoEnhancer	Plain and enhanced karyotype maps derived from CytoVision	Enhanced tumor karyotype image	CycleGAN	(49)
Liu	2022	SRAS-net	Biological image chromosome classification dataset	Classification of low resolution chromosome images	SRAFBN, SMOTE (DL)	(12)
Xia	2023	KaryoNet	R-band and G-band clinical data sets	Simultaneously predict chromosome type and polarity	DL	(11)

CNN, convolutional neural network; CycleGAN, cycle generative adversarial network; DL, deep learning; SRAFBN, self-attention negative feedback networks.

Notably, most chromosome classification and karyotype analysis systems have certain limitations, such as low-quality and poor-resolution karyotype maps. These issues can easily lead to missed or difficult-to-prepare reports on breakpoints within chromosomes. To improve the clinical analysis of low-quality karyotype maps, several AI models have been explored in recent years. They can convert low-quality karyotype maps into high-resolution images by specific algorithms, so as to accurately detect hidden chromosomal abnormalities (49) or obtain clearer chromosomal features, with an accuracy of up to 97.55% (12). In addition, type and polarity classification can speed up the process of generating karyotype maps, but chromosomes are prone to bending in microscopic images. This hinders cytogeneticists from analyzing chromosome types. Chromosome classification and straightening based on interleaving and multitasking networks can make chromosome band information easier to read (13). To address this, Li et al. (14) developed a shielded conditional variational autoencoder for chromosome straightening. Combining this with other karyotyping systems can greatly improve the chromosome classification performance of various DL models, reduce the burden on clinicians, and save time and materials.

### 2.4 AI-assisted diagnosis of hematological disease in molecular biology

Molecular biology tests are another critical aspect of the comprehensive clinical diagnosis of hematological disease. Molecular testing of blood, including polymerase chain reaction, DNA sequencing, transgenics, and gene chip (DNA chip) technology, can predict and diagnose disease progression by testing various genes and their mutations in leukemia cells, particularly those that may lead to disease onset or regression.

With the introduction of high-throughput sequencing technologies such as multi-gene sequencing, whole-genome sequencing, whole-exome sequencing, and transcriptome sequencing, molecular biology has entered the realm of big data. These advanced sequencing technologies are now approved for clinical purposes and greatly facilitate the diagnosis of hematological disorders, ushering in the era of “precision diagnosis” for these diseases (Table 4).

**Table 4:** Application of artificial intelligence in molecular biology diagnosis of hematological diseases.

	Focus	Year	System	Input	Function	AI	References
Genomic analysis	Genome-wide data	2020	AML classifier	GEP	Risk prediction, differential diagnosis and subclassification of AML were performed	ML	(50)
		2020	AML classifier	AML cancer gene expression dataset	Predicting genetic associations in acute myeloid leukemia disease	OCSVM	(51)
		2021	ChINN	DNA sequence	Predict open chromatin interactions in DNA sequences	CNN	(15)
	Next-generation sequencing (NGS)	2017	WfG	Genomic DNA by NGS	Identify candidate driver mutations and related pathways	NLP	(60)
		2019	WfG	CS data	Identify candidate driver mutations and related pathways	NLP	(61)
	DNA methylation (DNAm)	2020	MethylNet	DNAm data	Discovery of unknown heterogeneity	DL	(58)
		2021	MethylCapsNet, MethylSPWNet	DNAm data	Capture characteristics associated with aging, cell type, and disease progression	DL	(55)
		2020	EpiScore	Single-cell RNA-seq tissue map	Quantitative specific differential methylation signals	DL	(56)
		2023	EpiScore	Single-cell RNA-Seq dataset	Facilitate the microanatomy of DNA methylation groups in large tissues	DL	(57)
Transcriptome analysis	Single-cell RNA sequencing	2016	SVM or RF classifier	Gene expression data of AML samples	Identification of characterized genes with the ability to predict FLT3/ITD mutation status	SVM, RF	(145)
		2019	BERMUDA	scRNA-seq date	Reduced batch effect	ML	(68)
		2021	RCA2	scRNA-seq date	Reduced batch effect	ML	(67)
Proteomic analysis	Protein pathways	2019	Stacked autoencoder	Proteomics data	Identification of key protein pathways involved in FLT3-ITD mutations	DL	(73)
	Fusion protein targets	2023	XG Boost Model	Integrated multi-omics datasets	Reveal the PML:: RARA gene target in APL	ML	(72)

AML, acute myeloid leukemia; ChINN, chromatin interaction neural network; CNN, convolutional neural network; CS, clinical sequencing; DL, deep learning; DNAm, DNA methylation; FLT3, FMS-like tyrosine kinase 3; GEP, gene expression profile; ML, machine learning; NGS, next generation sequencing; NLP, natural language processing; OCSVM, one-class classification support vector machines; RF, random forest; SVM, support vector machine; WfG, Watson for Genomics; XG Boost, extreme gradient boosting.

#### 2.4.1 Genomic analysis

Due to the sheer volume of data analyzed, annotation of clinically actionable genomic regions and their biological functions with prioritization of genetic variants is necessary for clinical decision-making and is the basis for automated disease classification, a process known as functional genomics. More research is being conducted to sequence a set of gene regions associated with a suspected disease, which not only reduces costs but also increases efficiency.

AML is a serious hematopoietic malignancy, and prediction of its status by whole-genome sequencing is essential for clinical diagnosis and treatment. Using deep neural networks of high-dimensional machine learning, combined with genomic analysis, it is now possible to subtype, differential diagnose, and predict risk for AML, while also obtaining meaningful predictive genetic signatures. The accuracy of this AML classifier is directly proportional to the number of samples, so the establishment of reference blood gene expression profiling data sets of a large number of samples is helpful to the obtained high-precision classifier, which can effectively solve the problem of lack of professionals in some regions or medical environments and greatly reduce the cost (50). Like most models, systems for predicting AML-associated genes are typically binary classification models that distinguish between known causative genes and unknown genes. A more accurate AML classifier focusing on known genes has been developed based on a one-class SVM (51). The reduced potential error rate compared to binary classification models demonstrates that gene expression profiling can provide more efficient, objective, robust, and prospective clinical guidance with the assistance of AI.

The importance of DNA methylation (DNAm) in cancer development and progression has been revealed in recent years (52). DNAm involves the addition of methyl groups to nucleotides without a change in the DNA sequence, and it most often occurs on cytosine–guanine dinucleotides. Hypermethylation of suppressor genes and hypomethylation of oncogenes in hematological malignancies such as MDS and acute lymphoblastic leukemia (53, 54) are part of the pathogenesis of these diseases and can lead to a poor prognosis. Traditional analysis of heterogeneity changes associated with phenotypes involves multiple-hypothesis testing and managing multiple covariates, which presents a significant challenge for genomic data analysis. However, with the development and integration of various AI algorithms and applications, DL has shown great potential and promising results for discovering and analyzing DNAm on actionable genomic targets. This advancement correlates with improved diagnosis of blood diseases (55). Teschendorff et al. (56) used DL algorithms to develop EPISCORE, a single-cell histology reference model that reduces the cost of inferring DNAm differences in patients with acute lymphoblastic leukemia by analyzing DNAm cell heterogeneity using high-resolution datasets (57). Another DL model, MethylNet (58), can uncover unknown DNAm through a combination of unsupervised generation, clustering, cell type deconvolution, subtype classification, age regression, and smoking status classification. It is also user-friendly.

With the increasing availability of genetic data, next-generation sequencing (NGS) has become a prerequisite for accurate diagnosis and proper treatment in clinical hematology/oncology (59). Watson for Genomics (WfG), a semi-automated pipeline AI system based on in-house NLP algorithms, has been developed for applications in clinical sequencing and precision medicine for patients with hematological malignancies. Tojo (60) used malignant and normal tissues from more than 150 patients as inputs and combined them with WfG to analyze the sequence data, identify mutations and associated pathways, and infer applicable drug information. Yokoyama (61) demonstrated the role of AI in the manual interpretation process of clinical sequencing using clinical sequencing data from more than 300 patients with hematological cancers as inputs.

The gold standard for hematology diagnosis has been challenged by the introduction of whole-genome sequencing and NGS combined with AI (62). AI shows the potential to outperform experienced hematologists in comprehensive analysis of high-dimensional whole-genome sequencing and NGS data, effectively narrowing interobserver variability and improving diagnostic timeliness and accuracy. In addition, precise and comprehensive AI-assisted genomic analysis is expected to be used more often to aid clinical decision-making. Because of the limited availability of genome-wide data, predicting chromatin interactions between open chromatin regions using only limited DNA sequence data is a major challenge. However, the ChINN provides a potential solution by identifying open chromatin interactions in a genome-wide manner. This was verified in samples from patients with CLL, exhibiting extensive heterogeneity in chromatin interactions of prognostic genes and thus suggesting differential expression of oncogenes (15).

#### 2.4.2 Transcriptomic analysis

RNA sequencing (RNA-seq) is an important method of transcriptomic analysis, which can reveal specific biological processes and the molecular mechanisms involved in the development of hematological disorders at a holistic level. RNA-seq is most commonly used to analyze differentially expressed genes, which is a challenge in the diagnosis of hematological neoplasms due to their heterogeneity. With the construction of dynamic RNA databases (63), the development of single-cell RNA-seq technology has significantly advanced. It is now possible to detect the underlying mechanisms of blood tumor heterogeneity and the biological behaviors of tumors to identify new potential targets in the clinical setting (64–66), providing deeper clinical insights. However, with the increasing amount of single-cell transcriptome data, there is a growing tendency to analyze data jointly, which can easily generate batch effects. RNA-seq, as a high-throughput sequencing method, is particularly sensitive to batch effects because of its high detection accuracy. Therefore, Schmidt et al. (67) established a model called RCA2, which helps reduce the batch effect while unifying single-cell data. The DL-based Batch Effect Removal using Deep Autoencoder (BERMUDA) can distinguish between different batches of small conditional RNA-seq data and different cell populations while removing the batch effect (68). This suggests that ML and DL of AI algorithms can optimize the transcriptomic analysis process and provide more objective and precise data for clinical transcriptional data analysis.

#### 2.4.3 Proteomics analysis

The mining of protein variants from multivariate data is also an important part of the diagnosis of blood disorders. Increasing numbers of studies are exploring the clinical applications of proteomics technologies for monitoring treatment responses, disease progression, and microscopic residual foci, with initial results showing promise (69). Typically, proteomics studies involve thousands of observations and thus require preprocessing of data (70). ML techniques have been applied in proteomics analysis because of their powerful ability to categorize unknown samples (71). Among these techniques, SVMs have proven particularly effective for proteomics classification. In 2023, using a multi-omics dataset and an ML algorithm, a new fusion protein target called PML::RARA was reported. This target directs the mechanism of different features of the transcriptional response, confirming that disruption of the coagulation cascade response due to high expression of this gene is associated with acute promyelocytic leukemia coagulation dysfunction and hemorrhage, presenting significant clinical challenges (72). In addition, ML can aid the extraction of hierarchical features to identify key protein pathways in *FLT3* internal tandem duplication mutations in patients with AML (73). Overall, with the increasing popularity of proteomics and the generation of large amounts of proteomics data, the future challenge lies in utilizing AI to process these big data effectively. The goal is to discover and analyze new actionable targets or pathways to facilitate the diagnosis and treatment of blood disorders.

## 3 Application of AI in precision therapy for hematological disease

### 3.1 Biomarker recognition

During the last decade, a large amount of molecular biology information has been mined, and ML algorithms have enabled the identification of biomarkers in the body fluids of patients with hematological disorders. These biomarkers can be used for early diagnosis, disease typing, and prognostic assessment, greatly broadening the horizons of hematological diagnosis. This advancement has deepened the understanding of the complex interactions among different molecular subgroups in hematological disorders, helping clinicians make precise estimations of interindividual variability and providing a reliable basis for subsequent targeted therapeutic regimens. This approach helps clinicians accurately estimate variability among individual patients and provides a solid foundation for developing targeted therapeutic programs (74). In addition, methods that incorporate ML create correlations between different layers of information, are user-friendly across various platforms, and enable the sharing of resources.

A few studies have relied on ML to discover or utilize new biomarkers for blood disorders. For example, Venezian Povoa et al. (75) used clinical and molecular data to predict treatment sensitivity in patients with MM and found that using gene expression values as markers improved the accuracy of predicting treatment sensitivity. In addition, combining high-throughput histology data with clinical data demonstrated higher clinical utility. Ghobadi et al. (76) characterized a variety of mRNAs and miRNAs in asymptomatic carriers of adult T-cell leukemia/lymphoma, identifying reliable miRNA–mRNA interactions for each subtype. These interactions serve as potential targets for subsequent therapy and as biomarkers for prognosis. Guerrero et al. (77) integrated cytogenetics [*t*(4; 14) and/or del (17p13)], tumor load (clonal size of bone marrow plasma cells and circulating tumor cells), and immune-related biomarkers to develop efficient, integrated weighted models for predicting undetectable measurable residual disease in MM episodes. Immune biomarkers showed the highest weight in these models, suggesting their importance in predicting the measurable residual disease status.

The potential of ML to aid the discovery of new biomarkers provides an opportunity to develop new AI systems that alleviate the pressure on clinicians to deal with massive amounts of data. At the same time, prognostic indicators based on ML-derived patient characteristics provide a more objective assessment of potential risk, facilitating the creation of clinically targeted therapies for new biomarkers and enabling personalized treatment for patients (74).

### 3.2 Drug development

Drug discovery and development is a long and costly process. During the past decade, ML, DL, and NLP have emerged as breakthrough technologies, accelerating the process because of their automated nature, predictive capabilities, and expected efficiency gains (78).

The persistence of leukemia stem cells has been shown to lead to acute leukemia treatment resistance. Li et al. (79) combined multiple ML algorithms to identify signatures specific to the gene expression of leukemia stem cells. Zhang et al. (80) found that genes associated with leukemia stem cells, particularly *RFC4* and *RFC5*, exhibit strong cluster interactions and may serve as inhibitory therapeutic targets for AML. ML can analyze large amounts of high-quality molecular biology data to identify targets of drug resistance during treatment. By discovering drug–target interactions, it can predict therapeutic efficacy, aid the design of new drug molecular structures, optimize drug efficacy, and help minimize side effects. CNNs in particular excel in image analysis, aiding biomarker identification and optimizing drug formulations. An example of this is AlphaFold, a CNN-based protein structure prediction and drug formulation system developed by DeepMind (Google). This neural network-based system excels in protein structure prediction and drug repurposing, significantly advancing the field of drug discovery and development (81).

Analyzing high-dimensional data to predict interactions between ligands and proteins is a difficult task in the drug-discovery process. Janssen et al. (20) developed a fully open-source drug-discovery mapping model that predicts the activity of novel kinase inhibitors in the kinome, such as the new inhibitor of *FLT3*. Continued progression and relapse of CLL after treatment remains problematic in patients with CLL that have been treated with new targeted agents (ibrutinib and venetoclax). Given the important role of microenvironment interactions in the progression and relapse of CLL, Gimenez et al. (82) built a drug-discovery platform to elucidate the critical role of simvastatin in targeting the microenvironment and its synergistic enhancement with ibrutinib or venetoclax for the treatment of CLL. These research endeavors suggest that ML algorithms can be used to build AI platforms that aid the development of new medicines or new combination therapies for blood disorders.

Furthermore, in addition to R&D to predict drug–target interactions and design new drugs, understanding the side effects of drugs and optimizing drug efficacy are aspects that cannot be ignored. Eltrombopag, a thrombopoietin receptor agonist for the treatment of primary immune thrombocytopenia, has side effects that affect systemic immunomodulatory responses. Therefore, Lozano et al. (83) developed the Therapeutic Performance Mapping System using AI pattern recognition to reveal the association between the target markers of eltrombopag (BCL1L2, BCL2, and BAX) and the key proteins involved in immune thrombocytopenia. This system has advanced our understanding of the drug’s mechanisms of action and side effects, providing valuable insights for drug optimization research and pointing to future directions for improving therapeutic efficacy and safety.

In summary, the incorporation of AI optimizes all aspects of drug development. It integrates with genomics, proteomics, and metabolomics to detect and dynamically monitor the effects of drugs, whether alone or in combination with other drugs. This greatly improves safety and efficacy outcomes and is expected to enhance patient prognosis and drive drug innovation. However, the use of AI technologies also faces a series of ethical and moral issues, such as data privacy and security, algorithmic bias and interpretability, and obtaining informed consent. Therefore, human oversight of intelligent algorithms is essential, and relevant laws and regulations need to be established or improved to address these challenges (84).

### 3.3 Disease prediction and risk stratification

Failure to treat hematological diseases in a timely manner is likely to be life-threatening. Malignant hematological diseases such as AML, MDS, and myeloproliferative neoplasms progress relatively rapidly and generally have a poor prognosis. However, previous studies have demonstrated that specific changes in these disorders can be detected prior to diagnosis (85). By analyzing a patient’s genomic data and lifestyle factors, AI can quickly and accurately predict the risk of developing a particular blood disorder, helping physicians to intervene early. Mahmood et al. (86) reported that using multiple supervised ML algorithms, it is possible to predict a significant risk of pediatric acute lymphoblastic leukemia based on clinical variables, phenotypic data, and environmental factors to inform treatment and prognosis. Nazha et al. (87) also demonstrated that stochastic survival algorithms combined with multidimensional data can facilitate dynamic risk stratification and personalized prediction of overall survival in patients with MDS. This ability of ML algorithms to quantify the risk of various myeloid tumor subtypes and make dynamic predictions can certainly provide more convenient one-stop prediction tools for clinical use (85).

A variety of ML algorithms can be employed to develop predictive models to optimize the rational allocation of clinical treatments, such as SVMs, artificial neural networks, RFs, decision trees, logistic regression, and k-nearest neighbors (88). However, most of these algorithms have limitations that are difficult to explain to clinicians. Consequently, recent studies have increased the use of explainable AI (XAI) methods such as Local Interpretable Model-agnostic Explanations (LIME) and Shapley Additive Explanations (SHAP), which are designed to help clinicians to more intuitively understand the impact of risk factors (89, 90). For immunocompromised patients with hematological malignancies, the SARS-CoV-2 mRNA vaccine is not effective and may increase the risk of severe breakthrough infections. According to Rodríguez-Belenguer et al. (91), ML algorithms (particularly SVM, which shows the best performance) and XAI can efficiently select patient-specific features to enhance predictions and improve healthcare strategies, which makes the widespread use of AI systems in the clinical setting more promising.

### 3.4 Individualized treatment and prognosis

With the continuous accumulation and expansion of systems biology data, including molecular biology, genomics, proteomics, metabolomics, and bioinformatics, along with precise estimation of interindividual variability and ongoing efforts to establish correlations between different layers of information, improved targeted therapeutic regimens are increasingly emerging in clinical practice. It is evident that the demand for both precision medicine and personalized medicine is gradually increasing among both doctors and patients. AI holds significant promise in promoting personalized medicine by enhancing the accuracy and efficacy of these tailored therapeutic approaches (92). Previous studies have demonstrated that, in conjunction with systems biology data, AI-automated networks can generate dynamic data clouds that are unique to each patient. These data clouds help physicians understand disease mechanisms, identify relevant blood biomarkers, and define druggable molecular targets. Then this information can be used to develop individualized treatment regimens, including the selection of the most effective drug and determination of the optimal dosage (93).

AI-assisted individualized treatment and prognostic prediction models are widely used in patients with leukemia. Previous studies have shown that AI models based on ML algorithms can identify key prognostic molecular markers for AML using high-throughput sequencing expression profiling data (94). These models can also predict complete remission or event-free survival, calculate prognostic indices, and stratify survival for individualized prognostic prediction (95, 96). For pediatric patients with acute lymphoblastic leukemia receiving cranial radiotherapy, ML algorithms can also predict treatment efficiency by stacking integrated classifiers, helping physicians to track the patients’ status (97). Notably, most current predictive models are trained with multi-source data from a single patient, which facilitates more accurate and personalized treatment for that particular patient. However, developers should note that in-depth gene sequencing is rare in routine clinical diagnosis. If an ML model is trained on genomic data alone and lacks clinical variables, the stability and generalizability of the resulting AI model will be greatly reduced (95).

Despite the gradual application of targeted therapies with innovative treatments for leukemia, conventional treatment is still usually performed using allogeneic hematopoietic stem cell transplantation (allo-HSCT) (98). Such treatment carries risks of high therapeutic toxicity, infectious complications, graft-versus-host disease (GVHD), graft failure, and relapse. ML has been used to predict risk factors for death in patients treated with allo-HSCT (99), with alternate decision trees showing great potential (100, 101). This allows the analysis of multiple factors simultaneously and ensures the generalization and high accuracy of the results. In addition, the output of alternate decision trees has the advantage of visualization, which lays the foundation for extended clinical use (100). Among the many prognostic risks of allo-HSCT, GVHD is the top priority. Recent studies have utilized decision tree tools to stratify post-transplant survival and have successfully identified seven unique phenotypes of patients with chronic GVHD (101).

Moreover, several new prognostic scoring systems have been proposed for the treatment of myelofibrosis over the last 2 years, aiming to improve the identification of patients with the poorest prognosis following treatment with ruxolitinib and to increase the accuracy of patient stratification. Notably, two systems, the Artificial Intelligence Prognostic Scoring System for Myelofibrosis (AIPSS-MF) and Response to Ruxolitinib After 6 Months (RR6), have been developed (102, 103). Both systems outperform traditional models in stratifying patients, enabling early identification of patients with myelofibrosis who have impaired survival after ruxolitinib treatment. This advancement provides a foundation for personalized AI-based prognostic models.

New candidate biomarkers for disease progression, treatment response, and chemotherapy resistance in MM have been identified by mass spectrometry-based proteomics (69). The proteasome influences tumor development through tumor signaling pathways, immunomodulation, and drug resistance (104), with the ubiquitin proteasome pathway playing a crucial role in the development of MM. Ren et al. (105) constructed a Ubiquitin Proteasome Pathway Risk Score (UPPRS) system that combines nine genes related to the ubiquitin proteasome pathway. This system stratifies MM patients and utilizes the International Staging System to efficiently predict overall survival. In addition, a prognostic column chart was produced to uncover mechanisms of resistance to proteasome inhibitors. This can be used to identify new targets for inhibiting the ubiquitin proteasome pathway, aiming to reduce the recurrence rate and increase the cure rate of MM (105). Furthermore, an ML-based decision support system can identify proteomic profiles to provide sensitive/resistant chemotherapeutic agents for patients with MM, offering a more precise approach for personalized treatment (106).

XGBoost models, based on an ML gradient boosting framework, can more accurately represent nonlinear relationships between multiple features and outcomes than traditional linear models. This advantage was recently validated in the comprehensive prognosis of mantle cell lymphoma (107). Despite the success of these AI models in individualized treatment and prognosis of hematological disorders, they still have limitations. These include a lack of external validation cohorts and generalization issues, which need to be addressed in further research. To improve these models, future studies should focus on creating more standardized training samples and integrating multiple features from multi-omics and clinical measures. This integration will enhance the accuracy of prognostic predictions (Table 5).

**Table 5:** Application of artificial intelligence in the precision treatment of hematological diseases.

Focus	Year	Disease	Model	Function	AI algorithms	References
Identification of biomarkers	2020	AML	MCFS, IFS, SVM, RIPPER	Identify LSC-specific gene expression signatures	ML	(79)
	2021	Leukemia	LSC gene network	Analyze LSC gene transcriptional correlations and interactions between LSC proteins	WGCNA	(80)
	2021	MM	MuLT	Examine the predictive value of MM heterozygosity TS	Supervised learning	(75)
	2022	ATLL	SVM-RFECV	Classify different ATLL subtypes of AC	ML	(76)
	2022	MM	Synthetic weighted models	Predicting undetectable MRD in multiple myeloma episodes	ML	(77)
Drug development	2019	AML	DDM	Predicting novel inhibitors of FLT3	t-SNE	(20)
	2020	CLL	ANN model	Identify drugs that target key proteins that function in the microenvironment	ANN	(82)
	2021	ITP	TPMS	Obtaining a more rational pathway between eltrombopag target and key ITP proteins	ML	(83)
Prediction of disease	2020	ALL	CART, RF, GM, C5.54 decision tree	Identify significant risk for ALL	ML	(86)
	2023	AML, MDS, MPN	MN-predict	Dynamic prediction of myeloid tumor risk	Cox regression	(85)
Individualized treatment and prognosis	2019	AML	Prediction model based on nomogram	Identify key prognostic molecular markers of AML and predict prognosis	Cox regression	(94)
	2019	AL	Prediction model of relapse after allo-HSCT	Aids decision-making in allo-HSCT	ADTree	(100)
	2019	AML	ANN model	Improves stratification accuracy while identifying strong predictors of AML survival	ANN	(96)
	2020	ALL	Stacked ensemble classifier	Predicts CRT therapy in pediatric ALL patients	ML	(97)
	2021	MDS	Prognostic models	Dynamically predict survival, probability of leukemia transformation and risk stratification in MDS patients	Stochastic survival	(87)
	2022	MF	RR6	Early identification of survival-impaired MF patients who may benefit from timely treatment conversion to RUX therapy	ML	(103)
	2023	AML	A multi-stage ML decision model	Predict and risk stratify complete remission and survival in AML	ML	(95)
	2023	MF	AIPSS-MF, RR6	Improve ability to identify subgroups of worst patients for stratification	ML	(102)
	2023	MM	UPPRS	Assess associations between clinical outcomes and PI and UPPRS-triggered responses	Cox regression, LASSO	(105)
	2023	MCL	XGBoost	Accurately predict MCL disease outcomes in large patient cohorts	ML	(107)

AC, asymptomatic carriers; ADTree, alternate decision tree; AIPSS-MF, artificial intelligence prognostic scoring system for myelofibrosis; AL, high-risk acute leukemia; ALL, acute lymphoblastic leukemia; allo-HSCT, allogeneic hematopoietic stem cell transplantation; AML, acute myeloid leukemia; ANN, artificial neural network; ATLL, adult T-cell leukemia/lymphoma; CART, classification and regression tree; CLL, chronic lymphocytic leukemia; CRT, cranial radiotherapy; DDM, drug discovery map; GM, gradient lifting machine; IFS, incremental feature selection; ITD, internal tandem duplication; ITP, primary immune thrombocytopenia; LASSO, least absolute shrinkage and selection operator; LSC, leukemia stem cells; MCFS, Monte Carlo feature selection; MCL, mantle cell lymphoma; MDS, myelodysplastic syndrome; MF, myelofibrosis; ML, machine learning; MM, multiple myeloma; MPN, myeloproliferative neoplasm; MPL, thrombopoietin receptor; MuLT, multi-learning training method; OS, overall survival; PI, proteasome inhibitor; RR6, response to ruxolitinib after 6 months during ruxolitinib treatment; RIPPER, repeated incremental pruning to produce error reduction; RUX, ruxolitinib; SVM, support vector machine; SVM-RFECV, support vector machine recursive feature elimination and cross validation; TS, treatment sensitivity; t-SNE, t-distributed random neighbor embedding; TPMS, treatment effect mapping system; UPPRS, ubiquitin-proteasome pathway; WGCNA, weighted gene co-expression network analysis.

## 4 Difficulties and limitations of AI-assisted applications

Optimizing standardized clinical care for hematological diseases is a crucial and challenging area of research. Standardized care in hematology involves providing uniform treatments for patients based on clinical practice guidelines and best practices to achieve optimal outcomes and consistent patient care regimens, reducing variability due to differences in medical practice. The development of standardized treatments is typically based on extensive clinical research and experience to determine the most effective and safest treatments for specific situations. These guidelines and specifications are often developed by professional medical organizations, academic institutions, or government agencies.

In the past 5 years, the rapid advancement of emerging technologies such as AI, ML, and DL has led to the development of a series of AI applications based on generalized comprehensive diagnosis and treatment. These applications have been widely used throughout the entire process of managing hematology patients, from prediction and diagnosis to treatment and prognosis. They range from analyzing peripheral blood smears and bone marrow smears to identifying genome heterogeneity and molecular biomarkers.

The integration of AI and human expertise is effectively advancing the intelligence, standardization, and normalization of hematology diagnosis and treatment. AI algorithms help humans analyze and interpret vast clinical data, improving the efficiency, objectivity, and accuracy of diagnosis and guiding clinical decision-making, particularly for inexperienced doctors. In addition, they can integrate data from multiple sources and uncover potential new clinical discoveries, thereby extending human capabilities and deepening knowledge and understanding of hematological disorders. This integration opens new directions and pathways for future research and improvement of treatment prognoses. In essence, AI enhances our understanding of blood diseases and offers new opportunities for improving therapeutic outcomes.

**Figure 3:**
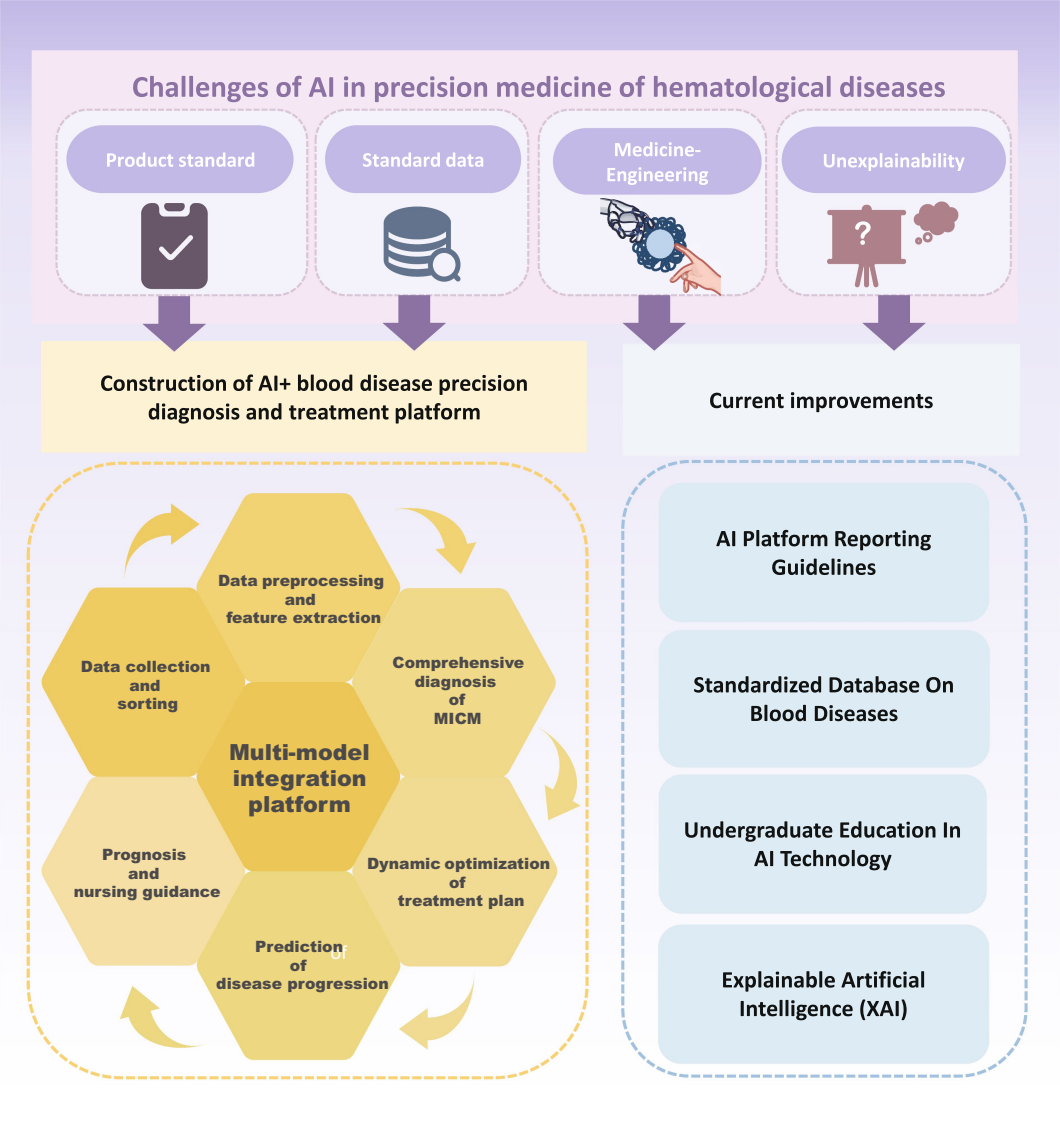
Challenges and future improvement of artificial intelligence in precision medicine of hematological diseases.

However, there are many difficulties and shortcomings in the process of using AI to these ends (Figure 3). First, any AI product needs to obtain a medical device registration certificate before it can be used clinically, which means there needs to be a standardized AI product that has been comprehensively validated. This is a prerequisite for integrating AI models into clinical practice. In terms of morphological recognition, many digital image analyzers using AI algorithms have been developed to automate blood smear examination and pre-classify cells. However, these come from different manufacturers and exhibit variation in staining methods, optical magnification, color, and display characteristics, hardware, software, and file formats. This lack of standardization presents significant challenges for consistent and reliable clinical application (108). The lack of standardization of parameters throughout the analysis process could also somewhat limit the extended use of this technology. In addition, because AI undergoes continuous ML to improve its performance, the integration and training of new data during the ML process is dynamic and flexible. This means that the system’s heterogeneity is susceptible to analytical confounders that do not align with highly stringent clinical trial standards and regulations. These confounders include batch effects, interinstitutional differences in sample collection, and pre-analytical processing protocols, which lead to difficulties in comparing and evaluating new algorithms (109). Therefore, the incorporation of AI technology does not mean the complete liberation or exclusion of humans. Instead, it emphasizes the need for human-supervised use to assist in clinical diagnosis and treatment. It also highlights the importance of establishing comprehensive guidelines and standard evaluation criteria for AI tools.

The second issue is the lack of standardized data and poor reproducibility. Data form the foundation of AI applications, and the large global population base provides an opportunity to establish extensive databases. It is widely acknowledged and confirmed that increasing the sample size greatly benefits algorithm performance enhancement by improving AI model accuracy. However, available training data for blood diseases is highly complex and limited, which makes it time-consuming to collect a large number of samples. During this process, changes in equipment, procedures, and personnel may occur, potentially leading to incomplete capture of morphological, genetic, or molecular biological variation, as well as susceptibility to issues such as overfitting and accidental fitting of confounding factors. In actual research, different laboratories and teams utilize various AI detection platforms with differences in model workflow, detection reagents, and selection parameters, which are constrained by infrastructure limitations. This results in uneven quality of similar data, which significantly reduces homogeneity (110). In addition, the relative independence of medical institutions makes it difficult to intercommunicate and share data, compromising the transferability of AI models across different clinical institutions. In the field of diagnosis and treatment of hematological diseases, systematic, and comprehensive high-dimensional data for ML are needed to provide accurate and robust prediction results to guide treatment strategies (95). Particularly when using ML for analyses involving sensitive and confidential patient data, a lack of standardization is likely to raise ethical issues regarding data privacy and security (111). Therefore, it is necessary to screen and construct effective datasets while complying with relevant regulations such as the EU General Data Protection Regulation. Strengthening the integration of laboratory information systems and electronic health record systems with ML, building a mature data-sharing platform, and establishing a comprehensive data standard system are crucial to ensure data security and maximum sharing. Using ML on this basis is more likely to promote data-driven implementation into all aspects of clinical care, such as accelerating the process of drug discovery and development and reducing failure rates for hematological diseases (81).

Another key difficulty is the lack of intersection between medicine and engineering. Given the complexity of ML, which requires continuous refinement of AI, clinicians and researchers often encounter several pitfalls when designing and developing new models (112). When constructing a model, the data are usually decomposed into a training set and a test set. In experiments, researchers often overestimate the actual effect of the model because of improper data splitting. Typically, only the target variables are changed during the experiment; however, the ML algorithm is highly sensitive to hidden variables, leading to confounding factors. It is necessary to use multiple ML models to detect these hidden variables. Data output from an AI model may be biased, and model developers may misunderstand the goal. Therefore, the rich experience of professionals and standardized diagnosis and treatment processes are crucial for improving the applicability of ML in the diagnosis and treatment of hematological diseases. However, the number of experienced and professional hematologists is currently limited, and few clinicians have knowledge of AI technology or are skilled in its development, research, and use. Cultivating such composite high-end talents requires a significant amount of time and economic cost. This limitation hampers the increasing effectiveness, precision, and clinical adoption of AI products.

The “black box” is an objective shortcoming of AI technology. AI relies on continuous ML to improve its performance. ML includes three components: algorithms, training data, and models. An algorithm is a set of procedures that, in ML, learns to recognize patterns after being trained on large amounts of data. Once an ML algorithm is trained, the result is an ML model, which can be used by people. DL models usually contain a large number of parameters and complex network structures, enabling them to handle high-dimensional data and learn complex feature representations. However, the complexity of the model also makes its inner workings unexplainable. Typically, any one of these three components can be hidden or figuratively described as being placed in a black box. To protect intellectual property and achieve higher accuracy and generalization, AI developers often obscure the model or the data used to train the model. This means that the training data are placed in a black box, which makes it impossible for non-AI professionals, such as clinicians, to directly understand how the model extrapolates decisions during the decision-making process. This difficulty ensuring the credibility and safety of the results has, to some extent, hindered the full integration of AI ML technology and clinical practice. In view of this, research in recent years has focused on the development of new XAI technologies (113–116), such as LIME and SHAP (89, 90). XAI can capture the results and outputs of ML/DL algorithms and provide model decision-making and interpretation to overcome the limitations of the black box nature of AI. XAI has shown great promise in diagnosis and prediction of drug discovery and development (116–118). However, XAI technology is still in the exploration and development stages and has not been effectively and comprehensively standardized.

Overall, AI and ML have demonstrated promising results in laboratory research and hold potential for widespread application in clinical auxiliary diagnosis and treatment. However, the lack of standardized product standards, uniform data, and high-level expertise hinders the transferability of AI and ML, leading to complexity and challenges in interpreting algorithms and models. The main obstacles that need to be addressed include establishing reasonable and legal clinical decision-making guidelines and enhancing the integration of AI into clinical practice.

## 5 Current research hotspots and suggestions

Due to the deepening reform and development of “AI + medical,” the field of blood disease diagnosis and treatment is undergoing major changes. Clinical laboratories are transforming the traditional purely manual comprehensive diagnosis and treatment model by integrating clinical decision support systems with AI, ML, and DL algorithms (119). This entails the integration of morphology, immunology, genetics, and molecular biology with advanced high-precision technology. Through data collection, sorting, preprocessing, feature extraction, model establishment, validation, evaluation, and treatment optimization, AI has significant applications across all stages of blood disease healthcare. These stages include early diagnosis, personalized treatment plans, disease progression prediction, real-time prognosis monitoring, and drug management. The implementation of AI greatly reduces the need for manual intervention while significantly improving efficiency and accuracy. This enables doctors to make more informed decisions in clinical practice and assists in promoting the standardization of clinical diagnosis and treatment.

Despite the great convenience brought by AI, there are still many challenges to integrating it into clinical practice, highlighting directions for subsequent research. First, it is necessary to fill the gap of specific criteria for AI-assisted diagnosis of hematological diseases and establish a standardized database. The classification of chromosomes in the karyotype analysis of patients with blood diseases is cumbersome and error-prone. Most of the existing AI models have been developed from different private datasets with poor transferability. To address this, a research team has established publicly available clinical chromosome classification datasets (120). Similarly, the LeukmiR genetic database was developed based on AI algorithms (63).

Second, the application of AI products needs to be evaluated in clinical trials, which requires the establishment of comprehensive reporting guidelines. These guidelines ensure standardized data output, address potential sources of bias specific to AI interventions, and enable integration into AI DL models. Ensuring the successful application and transferability of AI models across teams or laboratories is crucial. To address potential risks associated with AI use in the clinic, prospective evaluations involving AI interventions are necessary. Since the first reporting guidelines for clinical trials involving AI interventions (SPIRIT-AI) were developed in 2019 and the first international standards for clinical trials were jointly released in 2020 (121–126), the quality of clinical trials has improved. The latest guidelines indicate that in addition to the core items of CONSORT 2010, the CONSORT AI extension adds 14 new items (127), and the SPIRIT AI extension adds 15 new items (128). These additions address the processing of inputs and outputs, the setting of interventions, the interaction between humans and AI, and the analysis of error cases. This improves transparency and integrity in reporting clinical trials of AI interventions. In addition, the Minimum Information about Clinical Artificial Intelligence Modelling (MI-CLAIM) guidelines and the Minimum Information for Medical AI Reporting (MINIMAR) series have been released. These guidelines are designed to help researchers improve their designs and model quality (121). It is believed that more comprehensive guidelines will be developed to evaluate and regulate the clinical application of AI products in the future.

In addition, with the rapid development of AI technologies such as ChatGPT and their application in the field of education, humans are gradually moving toward a new path of collaborative development and symbiosis with AI in education (129). Similarly, the collaboration between medical professionals and AI is becoming essential for the advancement of AI in healthcare, ensuring that AI can effectively adapt to and enhance medical treatment. The current clinical and research environment suggests an urgent need for more clinicians with strong professional abilities to participate in the research and development of AI systems. This involvement will help replicate and promote the experience of doctors, produce more AI products, and ensure that these technologies are closely aligned with clinical needs to better serve patients. Accelerating the use of AI and clinical decision support systems (AI-CDSSs) in clinical practice is essential. However, there is a shortage of high-end composite talents in the field. According to international surveys, undergraduate medical students show a strong interest in AI and have high expectations but most have not received relevant education. They often do not understand the basic principles, limitations, and potential biases of various ML algorithms (130–133). However, large AI language models such as ChatGPT (129) can be used as virtual teaching aids to provide students with personalized and immediate medical knowledge and conduct interactive simulation learning and detection. Therefore, significant changes will gradually take place in the field of medical education. Increasingly more undergraduate medical colleges are beginning to focus on training students in AI technology. This includes providing personalized learning experiences, enhanced diagnostic training, and risk-free practical experiences (134). The School of Medicine at the University of Paris has introduced an elective course on AI-CDSS. This program allows medical students to assume the role of AI-CDSS designers and develop projects from six key aspects: determining needs, defining goals, designing educational strategies, implementation, formulating assessments, and designing project evaluations. The aim is to cultivate digital health skills and critical thinking, preparing students to adapt to the rapidly evolving digital environment (135). Dartmouth’s Geisel School of Medicine also offers elective courses on AI and its clinical applications. These courses include a preclinical hematology module, which enhances students’ understanding of advanced AI algorithms and models (136). Emory University School of Medicine offers an elective course on web-based resources and related research papers for fourth-year medical students without a background in data science or programming. An analysis of student project reports from this course concluded that even 1 month of AI and ML education during the undergraduate period can significantly improve students’ confidence in their understanding and self-reporting of AI and ML concepts (137), preparing them for current and future developments and changes in practices. Integrating AI into education fosters team cooperation with open communication between medical students, clinical medical workers, and AI experts. This approach leverages the technical skills of AI experts, the rich experience of clinical workers, and the valuable opinions and expectations of medical students, ensuring that research and development are closely aligned with clinical practice and meet clinical needs and preferences. However, this type of medical education has not been widely promoted on a global scale, primarily because of the lack of teachers knowledgeable in AI and the absence of standard guidelines for its use. To promote the effective and responsible use of AI in the future, several recommendations have been proposed: ensuring the transparency of AI system development and deployment, resolving biases in AI algorithms to ensure educational equity, verifying the output of AI educational tools, protecting the privacy of input data, obtaining informed consent, promoting multi-party cooperation, training teachers in AI ethics, conducting important ideological education, continuously maintaining the performance of AI algorithms, establishing clear accountability systems, and improving usage standards (134). In conclusion, future doctors should follow current development trends and be taught to become key users of AI as early as possible. They should understand the principles and basic scope of various algorithms and models and be able to critically analyze and evaluate the data and suggestions provided by AI. This approach will inject fresh perspectives and active force into the future optimization and standardization of clinical diagnosis and treatment via AI (74).

With the increasing integration of AI and ML algorithms into medical education, people’s understanding of high-end technology continues to deepen, and their willingness to use AI technology continues to grow. However, alongside this enthusiasm, there are also fears and concerns about human judgment being gradually reduced and replaced by AI (138). However, in fact, most studies have selectively highlighted the advantages and clinical applicability of AI products, often leading to exaggerated results to meet expectations. In addition, there are ethical challenges related to patient privacy, inherent biases in algorithms, and the interpretability-associated shortcomings of AI. In the future, it is expected that AI technology will be able to integrate medical data from any data owner worldwide without violating privacy laws, thereby crossing the technological divide and driving precision medicine for blood diseases (139).

To better use AI to promote the standardized diagnosis and treatment of blood diseases, we can comprehensively analyze and compare the current AI-assisted technology platforms or models applicable to this field. By selecting the optimal AI system, we can build a comprehensive platform for standardized, AI-assisted diagnosis and treatment of blood diseases based on the clinical pathway of the diagnosis and treatment of blood diseases. Continuous ML updates can improve diagnostic accuracy, guide clinical treatment, recommend the best and most personalized treatments, and dynamically track and adjust treatment plans as the patient’s condition changes. The platform or model should adhere to the clinical pathway, including data collection and sorting, data preprocessing and feature extraction, diagnosis, dynamic optimization of treatment regimens, prediction of disease progression, prognosis, and nursing guidance. This approach will promote the standardization of diagnosis and treatment of blood diseases. Recent research has also proposed a future involving multi-model integrated interpretation of data, where information from cell morphology samples, genetic analysis, and other datasets is combined to form a human–AI feedback loop (108). This approach aims to provide an overall semantic understanding as well as thoughtful diagnostic prediction and interpretation (140). In this way, hematologists can be assisted in real time to make the best clinical decisions. Integrated AI tools combining different ML techniques can automate repetitive tasks, including the assessment of cell morphology, FC, genetic data, and genetic mutation data, integrating all diagnostic modalities for the same blood disorder (74). However, the development and use of such multimodal DL methods should be accompanied by the establishment of regulatory oversight, legal frameworks, and monitoring systems to ensure effective clinical disclosure and safe application of AI products.

## 6 Conclusion

AI based on ML and DL algorithms has been continuously applied to the comprehensive diagnosis and precision treatment in hematological diseases (Table 6). Its powerful data analysis capability can improve the speed of laboratory diagnosis while maintaining or even surpassing the accuracy of human diagnosis. Notably, some AI digital recognition systems offer high degrees of visualization, presenting analysis and diagnosis results to clinicians in an intuitive and visual manner. In treating blood diseases, issues such as drug resistance, GVHD in allo-HSCT, and relapse are common. With the integration of various clinical data and ML, AI has been able to uncover potential pathogeneses, drug resistance mechanisms, and new therapeutic targets, providing more accurate guidance for drug innovation, risk stratification, and prognosis tracking. Despite the lack of AI product standards, the absence of standardized ML data, the challenges posed by the intersection of medicine and engineering, and the difficulty of interpreting algorithm models, progress has been made by establishing digital databases of blood diseases, formulating laws and regulations on AI ethics and privacy supervision, integrating AI algorithm education into medical undergraduate programs, and developing interpretable AI models. In short, AI has the potential to markedly enhance the precision of clinical diagnosis and treatment of hematological diseases. However, the ultimate goal is not to replace humans with AI but to aid the standardization of clinical diagnosis and treatment through AI products. To further improve and standardize precise diagnosis strategies and the treatment of blood diseases in the context of “AI + medicine,” more research is needed to build an integrated intelligent diagnosis and treatment platform that aligns with clinical pathways.

**Table 6:** The application of AI in the diagnosis and treatment of various hematological diseases.

Diseases		Application	System	Purpose and effect	References
Leukemia	APL	Diagnosis	Mask R-CNN	Detection and classification of nucleated cells using example segmentation methods	(26)
			XG Boost model	Reveal the PML:: RARA gene target in APL	(72)
			The multi-stage DL platform	Automatically reads bone marrow smear images, accurately segments cells, predicts APL	(27)
	AML	Diagnosis	FRCNN, VGG Image Annotator, ENN, Xception CNN, ResNet50	Distinguish AML and predict the mutational status of NPM1	(34)
			SVM or RF classifier	Identification of characterized genes with the ability to predict FLT3/ITD mutation status	(145)
			AML classifier	Risk prediction, differential diagnosis and subclassification of AML were performed	(50)
				Predicting genetic associations in acute myeloid leukemia disease	(51)
			ABMILM	Automatic diagnosis of AML and molecular characterization	(40)
			Cross-panel sample-level classification model	Diagnosis and classification of AML	(41)
		Treatment	A multi-stage ML decision model	Predict and risk stratify complete remission and survival in AML	(95)
			ANN model	Improves stratification accuracy while identifying strong predictors of AML survival	(96)
			DDM	Predicting novel inhibitors of FLT3	(20)
			MCFS, IFS, SVM, RIPPER	Identify LSC-specific gene expression signatures	(79)
			Prediction model based on nomogram	Identify key prognostic molecular markers of AML and predict prognosis	(94)
	ALL	Diagnosis	ALL Detector (ALLD)	Distinguishing ALL patients based on primary cellular micrographs	(142)
		Treatment	CART, RF, GM, C5.54 decision tree	Identify significant risk for ALL	(86)
			Stacked ensemble classifier	Predicts CRT therapy in pediatric ALL patients	(97)
	CLL	Treatment	ANN model	Identify drugs that target key proteins that function in the microenvironment	(96)
	Leukemia	Diagnosis	YOLOX-s, MLFL-Net	Cellular detection, classification and prediction of leukemia types	(30)
			Techcyte	WBC identification and vesicle recognition	(37)
			Faster R-CNN	Automatically detect bone marrow cells and determine their type	(25)
			CNN Model	Recognize all subtypes of leukemia	(32)
			ANN + FFNN + SVM^1^	Early detection of leukemia	(33)
			AlexNet, GoogleNet, ResNet-18^2^		
			CNN-SVM ^3^		
		Treatment	LSC gene network	Analyze LSC gene transcriptional correlations and interactions between LSC proteins	(80)
Lymphoma	MCL	Treatment	XGBoost	Accurately predict MCL disease outcomes in large patient cohorts	(107)
	ATLL	Treatment	SVM-RFECV	Classify different ATLL subtypes of AC	(76)
Myeloma	MM	Treatment	MuLT	Examine the predictive value of MM heterozygosity TS	(75)
			Synthetic weighted models	Predicting undetectable MRD in multiple myeloma episodes	(77)
			UPPRS	Assess associations between clinical outcomes and PI and UPPRS-triggered responses	(105)
Myelodysplastic syndrome	MDS	Diagnosis	DenseNet, YOLO	Detection and classification of cellular and non-cellular objects in samples	(140)
			BMSNet	Evaluation of single-nucleated sphere morphology in bone marrow smears	(29)
			Flow cytometry diagnostic system for MDS	Distinguish between MDS and non-neoplastic cytopenia	(144)
		Treatment	Prognostic models	Dynamically predict survival, probability of leukemia transformation and risk stratification in MDS patients	(87)
Myeloproliferative disorders	MF	Treatment	RR6	Early identification of survival-impaired MF patients who may benefit from timely treatment conversion to RUX therapy	(103)
			AIPSS-MF, RR6	Improve ability to identify subgroups of worst patients for stratification	(102)
	MPN	Diagnosis	Single Shot Multibox Detector	Determine megakaryocyte cytomorphologic subtypes and correlate extracted features with potential diagnosis of MPN or reactive/non-tumor mimics	(38)
Immune hematologic disorders	ITP	Treatment	TPMS	Obtaining a more rational pathway between eltrombopag target and key ITP proteins	(83)
Metabolic blood disorders	HM	Diagnosis	Techcyte	Assessing the accuracy of WBC classification and primitive cell identification	(37)
Metastatic cancer of bone marrow	MCBM	Diagnosis	Morphogo	Identifying metastatic atypical cancer clusters and facilitating rapid diagnosis	(143)
AA, MDS, AML		Diagnosis	Recognition model constructed by image-net pre-trained model	Automatic differentiation of AA, MDS and AML based on bone marrow smears	(28)
AML, MM		Diagnosis	BMAsDCC	Detect and classify all non-neoplastic bone marrow cell components of DCC and tumor cells	(22)
AML, ALL, CML, CLL		Diagnosis	ResNet50	Automated analysis of bone marrow smears using only slide-level labels	(141)
AML, MDS, MPN		Treatment	MN-predict	Dynamic prediction of myeloid tumor risk	(85)

AA, aplastic anemia; AC, asymptomatic carriers; AI, artificial intelligence; AIPSS-MF, artificial intelligence prognostic scoring system for myelofibrosis; ALL, acute lymphoblastic leukemia; ANN, artificial neural network; AML, acute myeloid leukemia; APL, acute promyelocytic leukemia; ATLL, adult T-cell leukemia/lymphoma; CART, classification and regression tree; CLL, chronic lympholeukemia; CNN, convolution neural network; CRT, cranial radiotherapy; DCC, differential cell count; DDM, drug discovery map; DL, deep learning; ENN, ensemble neural network; FFNN, feedforward neural network; FLT3, FMS-like tyrosine kinase 3; FRCNN, faster region convolutional neural network; GM, gradient lifting machine; HM, hematological malignancies; IFS, incremental feature selection; ITD, internal tandem duplication; ITP, primary immune thrombocytopenia; LSC, leukemia stem cells; MCFS, Monte Carlo feature selection; MCL, mantle cell lymphoma; MCBM, metastatic cancer of bone marrow; MDS, myelodysplastic syndrome; MF, myelofibrosis; ML, machine learning; MM, multiple myeloma; MPN, myeloproliferative neoplasm; MuLT, multi-learning training method; NPM1, nuclear phosphoprotein 1; PI, proteasome inhibitor; RF, random forest; RIPPER, repeated incremental pruning to produce error reduction; RR6, response to ruxolitinib after 6 months during ruxolitinib treatment; RUX, ruxolitinib; SVM, support vector machine; SVM-RFECV, support vector machine recursive feature elimination and cross validation; TPMS, treatment effect mapping system; TS, treatment sensitivity; UPPRS, ubiquitin-proteasome pathway; WBC, white blood cells; XG Boost, extreme gradient boosting.
